# An integrated approach to historical population assessment of the great whales: case of the New Zealand southern right whale

**DOI:** 10.1098/rsos.150669

**Published:** 2016-03-16

**Authors:** Jennifer A. Jackson, Emma L. Carroll, Tim D. Smith, Alexandre N. Zerbini, Nathalie J. Patenaude, C. Scott Baker

**Affiliations:** 1 British Antarctic Survey, NERC, High Cross, Madingley Road, Cambridge, UK; 2 Scottish Oceans Institute, School of Biology, University of St Andrews, Fife, St Andrews KY16 8LB, UK; 3 World Whaling History, Redding, CA, USA; 4 National Marine Mammal Laboratory, Alaska Fisheries Science Center, NOAA Fisheries, 7600 Sand Point Way NE, Seattle, WA 98115-6349, USA; 5 Cascadia Research Collective, 218 1/2 W 4th Ave, Olympia, WA 98501, USA; 6 Instituto Aqualie, Av. Dr Paulo Japiassu Coelho, 714, Sala 206, Juiz de Fora, Minas Gerais, Brazil; 7 Collégial International Sainte-Anne, Montréal, Québec, Canada; 8 School of Biological Sciences, University of Auckland, Auckland 1010, New Zealand; 9 Marine Mammal Institute and Department of Fisheries and Wildlife, Hatfield Marine Science Center, Oregon State University, Newport, OR 97365, USA

**Keywords:** whaling, historical abundance, southern right whale, bottleneck, recovery

## Abstract

Accurate estimation of historical abundance provides an essential baseline for judging the recovery of the great whales. This is particularly challenging for whales hunted prior to twentieth century modern whaling, as population-level catch records are often incomplete. Assessments of whale recovery using pre-modern exploitation indices are therefore rare, despite the intensive, global nature of nineteenth century whaling. Right whales (*Eubalaena* spp.) were particularly exploited: slow swimmers with strong fidelity to sheltered calving bays, the species made predictable and easy targets. Here, we present the first integrated population-level assessment of the whaling impact and pre-exploitation abundance of a right whale, the New Zealand southern right whale (*E. australis*). In this assessment, we use a Bayesian population dynamics model integrating multiple data sources: nineteenth century catches, genetic constraints on bottleneck size and individual sightings histories informing abundance and trend. Different catch allocation scenarios are explored to account for uncertainty in the population's offshore distribution. From a pre-exploitation abundance of 28 800–47 100 whales, nineteenth century hunting reduced the population to approximately 30–40 mature females between 1914 and 1926. Today, it stands at less than 12% of pre-exploitation abundance. Despite the challenges of reconstructing historical catches and population boundaries, conservation efforts of historically exploited species benefit from targets for ecological restoration.

## 1. Introduction

Population assessments of the recovery of the great whales from exploitation have a long history [1], and over 50 years analysis methods have improved, along with the underlying population dynamics modelling framework used ([2] and references therein). Population assessments place current abundance and trends in the context of past exploitation, measuring current recovery relative to pre-exploitation baselines, and providing a view on the state of ecological restoration and rate of recovery of a population. Such reviews are critically useful for conservation management decision-making regarding the protection of species and their habitats. A ‘long view’ on pre-exploitation baselines can also protect against shifting baselines in public perception and conservation goals [3].

Assessments of most whale species have, however, long been hampered by a lack of historical data, for example on past catches, and patchy or absent contemporary knowledge, for example on life-history parameters, trends in abundance and spatial structuring of populations. Population growth is usually assumed to be directly density-dependent, although selection-delayed dynamic models may be more biologically realistic [4]. There have been some recent advances in baleen whale population modelling: Bayesian approaches (e.g. [5]) are now the norm for incorporating uncertainties in parameter estimates, and an increasingly integrative approach can accommodate multiple data sources and enhance the biological realism of the population models employed [6–8]. Integrations include the use of current genetic diversity to put a constraint on the minimum number of whales surviving exploitation (i.e. minimum number of maternal lineages surviving the exploitation bottleneck [6]), the inclusion of catch record uncertainty [9] and the direct incorporation of mark recapture models into the population dynamic framework, to inform population abundance and trend [10].

Many large whale populations (in particular bowhead, right and grey whales) have experienced centuries-long periods of exploitation, which are understood in broad temporal and geographical terms [11,12] but can be challenging to reconstruct accurately at the regional level, as most pre-modern whaling records are patchy (e.g. [13]) and many are expressed in terms of oil and baleen, requiring statistical treatment to convert into numbers of whales killed. For example, populations of southern right whales (*Eubalaena australis*) were subject to an intense ninteenth century commercial whaling campaign, which ranged across the Southern Hemisphere and decimated this previously abundant species both on its coastal winter calving grounds and offshore summer feeding areas. In 2001, the International Whaling Commission (IWC) conducted a global assessment of southern right whale recovery relative to initial (pre-exploitation) abundance prior to commercial whaling [14]. Abundance estimates were summed across recovering southern right whale populations and pre-exploitation abundance was back-calculated to 1770, using a historical catch series summed across each decade and ocean. While this analysis estimated recovery levels at approximately 20–25% in 2009 across the Southern Hemisphere populations, this global assessment could not address the regional impacts on southern right whale populations, subject to different intensities and patterns of whaling over, and prior to, the exploitation period [14].

Southern right whales show a form of migratory culture, whereby parents transmit preferences for migratory destinations to offspring during a prolonged period of parental care. In the case of the southern right whale, there is evidence for maternally directed learning of both winter nursing and summer feeding grounds which leads to genetic structuring at both ends of the species' migratory network [15–17]. In the Southern Hemisphere, 12 distinct southern right whale winter calving grounds have now been identified, which vary substantially in size and levels of geographical and genetic isolation [14]. Strong fidelity to relatively accessible wintering grounds in South Africa, Argentina, South Australia and the New Zealand sub-Antarctic regions means that abundance and trend are known from study sites within these regions [18–21], and all are significantly genetically differentiated from one another, suggesting demographic independence [15]. Addressing regional population differences as we do here can provide more precise estimates of the population history and the recovery level of southern right whales at the regional level.

Among all the right whale populations, the southwest Pacific region currently has the best resolved catch history, following in-depth reviews of whaling logbooks and fishery records for New Zealand waters and offshore pelagic whaling grounds [9,12,22]. Three winter calving grounds are located in the southwest Pacific: (i) New Zealand mainland, (ii) New Zealand sub-Antarctic islands, and (iii) coastal southeast Australia. The southern right whale was once widely distributed in New Zealand waters and known as *Tohora* by the indigenous Maori people, and the two New Zealand wintering grounds may have been used by one population with a large-scale migratory pattern [23]. Females gave birth and nursed calves in the coastal waters of mainland New Zealand during the winter, moving offshore during summer months to feed [22,24]. However, southern right whales were the target of whaling around New Zealand from as early as 1791, one of many fronts in a global hunt for right whales which began in the first millennium AD [11]. Between 35 000 and 41 000 southern right whales are estimated to have been killed in New Zealand waters between 1827 and 1980, a period during which log-book, import and landings records permit a reasonably accurate reconstruction [9]. Hunting was so intense that this species had virtually disappeared from the New Zealand region by the early twentieth century [9,22]. In the greater South Pacific region, a similar pattern of early exploitation eradicated right whales on their wintering grounds in southeast Australia [22] and westward around South Australia [25,26] from as early as 1805 [11].

In the aftermath of hunting, no southern right whales were seen in New Zealand mainland waters for over 35 years (1928–1963) [27]. Today, the species appears to be slowly recolonizing former mainland wintering grounds from a sub-Antarctic refugium where it persisted during whaling [28]. The contemporary population uses wintering grounds off both the sub-Antarctic Islands (principally the Auckland Islands) and mainland New Zealand, although it is not clear if this was the case historically [21]. This population was estimated to comprise 2200 whales in 2009 and is recovering at 7% per annum [29].

**Figure 1.**
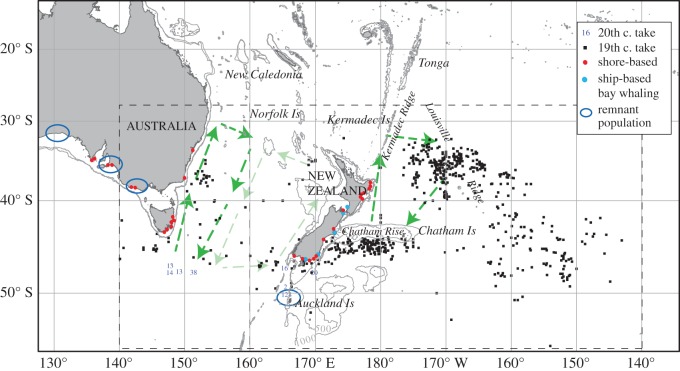
Occurrences of southern right whales in the southwestern Pacific. Whaling operations used in this study are shown by type and location as described by Carroll *et al*. [9] and Tormosov *et al*. [30]. Inferred seasonal offshore movements are represented by bold dashed arrows [22,23,25], while pale dashed arrows denote possible seasonal movements west of New Zealand. Regions encompassing historical catches considered in the current assessment are shown in the dashed boxes, with the left-hand box encompassing East Australian shore stations used in the ‘New Zealand plus East Australia’ high catch scenario. Figure modified from fig. 1 of Carroll *et al*. [9].

The offshore distribution of New Zealand southern right whales during summer and autumn is not well known. Historical whaling data suggest right whales were distributed widely at latitudes 35–50°S east and west of New Zealand, and particularly concentrated directly east of New Zealand (170°E to 175°W) and in the vicinity of the Kermadec Islands (figure 1) [9,12]. Migratory patterns inferred from whaling data also suggest that whales wintering off both New Zealand and southeast Australia may spend periods offshore in the Tasman Sea [12], with satellite tracking of right whales wintering in the sub-Antarctic Auckland Islands also revealing long-range westerly movements into high latitude waters south of Australia [31]. Catches of whales in these offshore regions are therefore likely to have been a mixture of whales from the waters of New Zealand and southeast Australia. There is also genetic evidence that individuals from these two regions might mix on shared migratory corridors [17]. The New Zealand population is genetically differentiated from the southwest Australian population, based on population structure and mating system studies [21,32]. In southeast Australia, the only area where southern right whales are now regularly seen with calves is Warrnambool, Victoria [33]. This population was estimated to number less than 250 individuals in 2014 [34]. There is greater differentiation seen between the two Australian wintering grounds than between either and the New Zealand wintering ground [21]. The level of differentiation between New Zealand and southeast Australia is low, and there are re-sightings of three individuals between South Australia and the Auckland Islands, indicating that there is some degree of on-going connectivity between New Zealand and South Australia [35].

Here, we reconstruct the historical demography of southern right whales in New Zealand waters, to quantify the impact of whaling and the level of recovery following protection [6]. Since both whaling and recovery levels will be impacted by the level of connectivity with neighbouring populations and offshore mixing, we encompass this uncertainty by developing catch scenarios to evaluate recovery. This study is the first in-depth reconstruction of the historical abundance of a southern right whale population, using catch reconstructions provided by analysis of whaling logbooks and import records [9] which incorporate uncertainty in the historical record, providing a framework for future population-level assessments, as well as insight into local recovery levels, critical for future conservation and management plans, and the historical role of the *Tohora* in the New Zealand ecosystem.

## 2. Material and methods

The contemporary New Zealand population of southern right whales encompasses winter calving grounds in New Zealand mainland waters, and an insular wintering ground on the New Zealand sub-Antarctic Islands, supported by multiple lines of evidence [17,28,32]. However, the offshore summer distribution of this population is largely unknown. Since both whaling and recovery levels will be impacted by the level of connectivity with neighbouring stocks and offshore mixing, this uncertainty has been encompassed through the development of two catch scenarios to evaluate recovery: (i) if New Zealand whales are only distributed in waters close to New Zealand and (ii) if New Zealand whales migrated further offshore, and could be hunted in waters off southeast Australia, termed the southwest Pacific catch series.

Population trajectories were reconstructed using an age- and sex-aggregated, density-dependent generalized-logistic model [36] implemented in a Bayesian framework so that uncertainty in estimates of abundance, trend and catch can be adequately incorporated [37]. Populations were assumed to be at pre-exploitation abundance (representing carrying capacity, parameter *K*) in 1827, prior to nineteenth century whaling. Data inputs for this model include:

(i) *Annual catch records.* We used the historical catch series' compilation for the southwest Pacific developed by Carroll *et al*. [9], and upwardly corrected catches to total numbers killed, using estimates of whales struck but lost from the fisheries. Annual variance in estimated catches is incorporated into the population model.
(ii) *Abundance and population growth data*. Mark recapture histories of 710 southern right whales, used within the population model as information on current abundance, and population growth. Data were based on individual identification from DNA profiles, derived from biopsy sampling of New Zealand sub-Antarctic Auckland Islands right whales from 1995 to 2009 [29]. Recaptures were fitted into the population assessment model to provide information about trend and abundance over this period.
(iii) *Bottleneck size* (*N*_min_) *and mtDNA haplotypes*. Constraints on the minimum abundance of right whales at the crux of the population bottleneck (*N*_floor_), derived from the number of maternally inherited mtDNA haplotypes sampled in each population and therefore surviving the bottleneck [38].

### 2.1. Annual catch records

Right whales in the southwest Pacific were hunted from the early nineteenth century by fleets of whaling vessels registered from New Zealand, Australia, Great Britain, America and France [22]. Catch records for New Zealand right whales were compiled from 1827 to the present day (figure 1):

(i) US pelagic whaling records were derived from the American Offshore Whaling Log-book collection. Mean numbers of right whales caught per voyage were multiplied by the number of American logbooks departing in that year that recorded whaling off New Zealand and off southeast Australia. These provide estimates of right whale catches and associated standard errors for each region (table 2 of [9]). Carroll *et al*. [9] calculated the average time frame over which American and French whaling ships operated in the focal areas following departure from port, enabling allocation of catches to years.
  American bay and offshore whaling operations were distinguished using selected log-book records, providing estimates of ships highly likely to have been bay whaling, and ships possibly bay whaling. Two bay whaling catch series were developed: a ‘low’ series including ships highly likely to have been bay whaling and a ‘high’ series including ships identified as possibly bay whaling [9].
(ii) Catches from French pelagic whaling in the southwest Pacific [39] were differentiated into offshore and bay whaling components by assuming the relative frequencies of catches made between whaling operations mirror those made by American whaling vessels [9].
(iii) New Zealand and southeast Australian landings of right whales were derived from shore-based whaling. Differences in primary sources available during the catch history review of Carroll *et al*. [9] motivated the development of two shore-based catch series, a ‘low case’ and a ‘high case’, which differ in estimates of whales landed over the most intense period of the fishery.
(iv) In the twentieth century, 294 southern right whales were killed by Soviet whalers [30] in waters south of New Zealand or in the vicinity of the Auckland Islands (allocated as ‘New Zealand’ catches), and an additional 78 whales were killed due south of Tasmania (allocated as ‘southwest Pacific’ catches). Locations are seen in figure 1.
(v) Upward corrections for whales struck but lost during shore-based bay whaling (iii) and pelagic whaling (components of (i) and (ii)) were made using log-book data [9]. Corrections were 1.27 (s.e. = 0.050) and 1.45 (s.e. = 0.054) for shore-based and pelagic whaling, respectively, and assumed to be normally distributed (see the electronic supplementary material, text S2).

Combining each of the low cases and the high cases for the bay and shore whaling operations provides a high and low right whale catch series for New Zealand waters and a high and low catch series for southwest Pacific waters, which encompass much of the uncertainty in the New Zealand catch record. Catch uncertainty was encompassed by building a prior catch series which incorporated the uncertainty in each data source, as described in the electronic supplementary material, text S2.

### 2.2. Abundance and population growth

Within the assessment model framework, capture–recapture data were used to populate an open population mark recapture model of similar parametrization to a Pradel model [40] with constant natural mortality and time varying capture probabilities.

Males and females differ in the patterns of usage of the calving grounds, with females returning on average every 3 years to calve, and males showing lower long-term site fidelity than females [29]. Therefore, we analysed recapture data for males and for females separately for each catch scenario, in order to avoid estimation bias due to sex-specific differences in recapture probabilities. Analyses conducted with female recaptures were considered primary analyses, with male recapture scenarios considered sensitivity analyses, since their resighting rates suggest lower long-term fidelity to the focal calving ground. Resightings data were directly integrated into the Bayesian age-aggregated population dynamics model (as described by Johnston & Butterworth [10]) and formed part of the likelihood function (see the electronic supplementary material, text S1). The function was adjusted so that the capture–recapture data were fitted to halved model-predicted abundance estimates, since the capture–recapture data were from one sex only and model-predicted abundances pertained to the whole population (see the electronic supplementary material, text S1). Within the model, annual survival was fixed to 0.97, similar to the long-term adult female survival values estimated by Best *et al*. [20] for South African right whales.

To test the sensitivity of results to the direct use of mark recapture data, we also constructed four scenarios where relative abundance and 2009 absolute abundance were fitted into the model instead of the mark recapture data, using estimates made with a POPAN model developed with male and female recaptures [29]. Model likelihoods were constructed as described by Zerbini *et al*. [37] for relative and absolute abundance measures, with the error distribution of both datasets assumed to be lognormally distributed. Relative estimates were *N* =  533, 619, 910 and 1074 for 1995, 1998, 2006 and 2009, respectively (CV = 0.2) and absolute abundance in 2009 was 2148 (CV = 0.20). Scenarios explored were high and low catch scenarios, two with *N*_floor_ = 36 and two with no bottleneck constraint.

### 2.3. Bottleneck size (*N*_min_) and mtDNA haplotypes

The number of mtDNA haplotypes sampled in each population was used to derive an estimate of the minimum number of female whales that could have survived the bottleneck [38]. The estimate of bottleneck size (*N*_min_) can thus be constrained by a lower boundary (*N*_floor_) based on the number of mtDNA haplotypes surviving in the remnant population. A recent survey of population structure [21] sequenced 12 mtDNA haplotypes from the Auckland Islands and mainland New Zealand, and 6 mtDNA haplotypes (all identical to Auckland Islands mtDNA lineages) from the southeast Australian wintering grounds, totalling 12 unique haplotypes region-wide. The number of mtDNA lineages present at the bottleneck represents the minimum possible population size for females at that time, assuming negligible impacts from subsequent genetic drift or migration.

MtDNA lineages were adjusted upward to account for males and non-contributing individuals at the bottleneck. The sex ratio was assumed to be 1 : 1, as observed from Auckland Islands surveys [41] and genetic identifications of calves [42].

In many wildlife populations only 10% of animals [43] are effective (i.e. give birth to offspring that survive to reproduce themselves). In order to accommodate the non-effective individuals in the population, we placed a conservative upward correction on minimum abundance from the 12 mtDNA haplotypes, where the number of haplotypes contributing (effective) females was assumed to represent at their maximum 33% of the population. Taking each haplotype as a proxy for a unique female, the number of haplotypes is multiplied by 1.5 to account for overlapping generations at the bottleneck (i.e. additional females not contributing unique haplotypes). This value is then doubled to account for males, to a final value of 3× the number of haplotypes in the extant population. The International Whaling Commission also use this ‘3×’ correction for constraining population models [44], since simulations of age-structured dynamics for Antarctic blue whales at their exploitation bottleneck suggest a minimum of 1.5 females per haplotype survive the bottleneck [45]. We also explored population outcomes when no minimum constraint on *N*_floor_ was imposed.

### 2.4. Population dynamics modelling

The population model structure is

$$N_{t+1}=N_{t}+N_{t}\cdot R_{\max}\cdot \left[1-{\left(\frac{N_{t}}{K}\right)}^{z}\right]-C_{t},$$

where *N_*t*_* is population abundance in year *t*, *K* is population carrying capacity in 1827, exponent *z* is fixed at 2.39 (corresponding to a maximum sustainable yield of 0.6 × *K*, as conventionally set by the IWC), *R*_max_ is the maximum population growth rate for the population, which is estimated when fitting the model, and *C_t_* is catches in year *t*.

A uniform prior was imposed on *N*_2009_ spanning 500–20 000 (this is intentionally broad and non-informative, and includes within its range current abundance values as presented in [29]). Upper and lower prior boundaries were chosen to span the full range of possible abundance, but to be narrow enough that computations could be optimized efficiently. A uniform prior was imposed on *R*_max_ spanning 0–0.12, to include a sufficiently wide range of biologically possible growth rates for southern right whales [20]. The prior distributions on *R*_max_ and *N*_2009_ were randomly sampled, and a unique catch series was generated by sampling from available data on pelagic and coastal catches and on struck and lost rates (see §2.1 and electronic supplementary material, text S2). A bisection approach was used to find the unique *K* corresponding to each set of prior values. The likelihood of each prior set (determining each trajectory) was measured using likelihood functions (electronic supplementary material, text S1). Only trajectories with abundance levels above the *N*_floor_ constraint were retained. The *N*_floor_ constraint therefore modifies the prior distributions before the data are added into the model. For the Importance Sampling approach for posterior inference, prior sets were weighted in proportion to their likelihood values. Fits were retained in proportion to likelihood using the Sampling-Importance-Resampling (SIR) algorithm as implemented by McAllister *et al*. [46], with 2000 resamples retained in the posterior distribution. For each prior set, population abundance was projected forward to 2020, in order to measure model-predicted abundance levels in 2015 and 2020 relative to *K*, a measure of population recovery.

To investigate the sensitivity of the mark recapture components of the model (male and female datasets) to different population growth rates, we picked 100 000 population growth (*R*) and abundance (*N*_1995_) values from uniform distributions of the form *R*[0,0.12] and *N*_1995_[36,2,100]. The *N*_1995_ distribution was bounded at the lower end by the 12 × 3 lower bound constraint on minimum abundance, and at the upper end by the estimate of super-population abundance for the population in 2009 [29]. Picks of *R* and *N*_1995_ were forward projected (*N_*t*_*_+1_ = *N_*t*_* + *N_*t*_* × *R*) to simulate exponential population increase rates from 1995 to 2009 (ROI_1995–2009_). The fit of each trajectory to the mark recapture model was calculated as a likelihood score (calculations in the electronic supplementary material, text S1), with annual survival fixed to 0.97 (see §2.2 for details). Parameters *N*_1995_ and *R* associated with the top 1% of likelihood values were plotted.

### 2.5. Population modelling scenarios

We constructed 16 population dynamic modelling scenarios, spanning multiple catch history, population identity, mark recapture abundance, trend and minimum population size scenarios.

Scenarios were designed to bound the likely catch history for the New Zealand population (table 1). However, considering likely overlap of the New Zealand and southeast Australian populations in the Tasman Sea and the uncertain origin of the landed whale oil, we developed multiple alternative catch histories to encompass the greatest possible impact on the New Zealand population. (1) A ‘New Zealand’ catch history, whereby all whales caught and landed in New Zealand waters, and whales caught by whaling ships which spoke for New Zealand were included. The offshore catch series were combined with either the ‘low’ or ‘high’ case New Zealand shore based and bay whaling catch series detailed in ‘Catch records’, forming two ‘high’ and ‘low’ New Zealand (‘NZ’) catch scenarios. While satellite telemetry indicates New Zealand right whales do travel outside this area [31], this scenario also assumes all whales east of 154°E are from New Zealand, so may include some wider-ranging whales from Australian calving grounds in turn. In the absence of information on offshore population mixing we therefore consider this a conservative catch allocation scenario. (2) A ‘Southwest Pacific’ (‘SWP’) catch history, where all American pelagic whaling off southeast Australia and New Zealand was allocated to the New Zealand population. Coastal catches estimated from returns and exports in New South Wales and Tasmania [22] were included in this scenario. This scenario provides an upper bound on the likely catch impact on the New Zealand population.

**Table 1.** Population identity, catch history and minimum abundance scenarios explored for New Zealand southern right whales. ‘NZ only’ refers to catches made in New Zealand sub-Antarctic and mainland waters. ‘SW Pacific’ refers to catches made in the southwest Pacific, including whales landed in east Australia and Tasmania. Details of the scenarios are given in §2.5.

scenarios	spatial extent of catches	Coastal NZ landings	NZ Bay whaling	*N*_floor_	abundance and trend
1	NZ only	low case	low case	0	female recaptures
2					male recaptures
3					fitted to POPAN relative abundance and N2009
4				36	female recaptures
5					male recaptures
6					fitted to POPAN relative abundance and N2009
7		high case	high case	36	female recaptures
8					male recaptures
9	SW Pacific	low case	low case	36	female recaptures
10					male recaptures
11		high case	high case	0	female recaptures
12					male recaptures
13					fitted to POPAN relative abundance and N2009
14				36	female recaptures
15					male recaptures
16					fitted to POPAN relative abundance and N2009

## 3. Results

**Figure 2.**
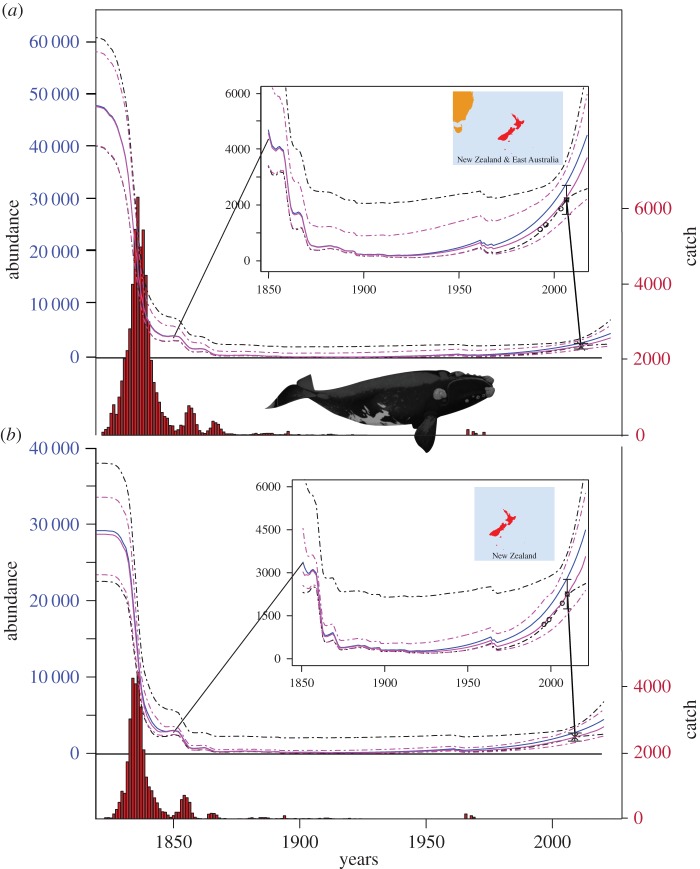
Population trajectories and catches of New Zealand southern right whales from 1829 to 2020. Panels show the population trajectories when (*a*) ‘high case’ catches from the southwest Pacific are allocated, and (*b*) New Zealand catches only, using the low case catch allocation. Median estimates are solid lines while dashed lines denote 95% probability intervals. Blue lines show the population trajectory when female recaptures are fitted. Pink lines show the trajectory fitted to relative abundance indices described in Carroll *et al.* [29]. Catch levels are shown as bar-plots beneath each population trajectory, with their frequencies plotted on the right-hand *y*-axis. Super-population abundance, calculated by Carroll *et al*. [29] for the Auckland Island population in 2009, is plotted as a cross with confidence intervals, while annual mark recapture estimates of numbers alive (obtained from the same model) are plotted as circles.

Our Bayesian population dynamic model provides the first estimates of the pre-exploitation abundance (*K*) of New Zealand right whales. We provide new insight into the steep decline and prolonged bottleneck due to 19th century exploitation, and slow population increase following illegal Soviet whaling (figure 2). As expected, backward projections of pre-exploitation abundance varied somewhat with the assumptions of the different catch scenarios. From the minimum (NZ low) to the maximum (Southeast Pacific high) catch scenarios, estimated pre-exploitation abundance increased by approximately 8000; rates of recent population growth (ROI_1995–2009_) and *R*_max_ (maximum possible rate of population growth) were not affected. Even with the most conservative catch scenario, however, pre-exploitation abundance was more than 28 000 in New Zealand waters, and perhaps more than 47 000 under the maximal scenario of all southwest Pacific catches allocated to this population (table 2).

**Table 2.** Posterior medians and 95% probability intervals for key biological parameters estimated for the NZ southern right whale over six population modelling scenarios with *N*_floor_ = 36. *K* refers to pre-exploitation abundance in 1829, *N*_min_ the estimated minimum bottleneck abundance, ROI, the rate of annual population increase, *R*_max_ the intrinsic rate of population growth. ‘max % depletion’ refers to the minimum abundance of this population during its exploitation history, relative to pre-exploitation abundance. ‘% recovery status' and ‘max % depletion’ show abundance as a proportion of pre-exploitation abundance *K,* in a given year and *N*_min_ year, respectively.

									abundance			% recovery status		
scenario	case	*R*_max_	*K*	*N*_min_	*N*_min_ year	max % depletion	total catch	ROI_1995–2009_	*N*_2009_	*N*_2015_	*N*_2020_	2009	2015	2020
female captures														
NZ only	low													
L 2.5%		0.005	22 505	38	1897	0.1	29 158	0.005	2083	2367	2523	6.2	6.8	7.1
median		0.045	29 156	111	1915	0.4	35 435	0.045	2723	3534	4428	9.4	12.2	15.2
U 2.5%		0.068	37 963	1986	1926	5.4	42 024	0.068	3596	4973	6749	13.9	19.6	26.3
NZ only	high													
L 2.5%		0.005	25 940	38	1897	0.1	34 067	0.005	2100	2346	2485	5.6	5.9	6.2
median		0.045	32 772	111	1915	0.4	40 347	0.045	2762	3578	4477	8.5	11.0	13.7
U 2.5%		0.069	42 075	1920	1926	4.8	46 993	0.068	3671	5151	7022	12.6	18.2	24.6
SW Pacific	low													
L 2.5%		0.004	35 823	38	1901	0.1	48 620	0.004	2121	2394	2549	4.2	4.6	4.7
median		0.048	43 231	106	1915	0.2	54 769	0.047	2752	3625	4575	6.4	8.5	10.7
U 2.5%		0.071	55 593	2042	1926	3.6	61 141	0.071	3685	5236	7147	9.2	13.6	18.8
SW Pacific	high													
L 2.5%#		0.005	39 549	38	1901	0.1	53 217	0.005	2122	2418	2554	3.9	4.2	4.4
median		0.046	47 100	114	1915	0.2	59 572	0.046	2758	3598	4499	5.8	7.7	9.6
U 2.5%		0.071	59 801	1993	1925	3.3	66 406	0.071	3639	5142	7040	8.4	12.3	16.8
fitted POPAN trend														
NZ only	low													
L 2.5%		0.016	22 905	38	1897	0.1	29 522	0.016	1517	1920	2269	5.0	6.1	6.9
median		0.048	28 784	91	1915	0.3	35 553	0.048	2236	2934	3673	7.8	10.2	12.9
U 2.5%		0.067	35 548	670	1926	2.1	41 929	0.067	3297	4438	5828	12.0	16.7	22.2
SW Pacific	high													
L 2.5%		0.013	39 636	38	1901	0.1	53 413	0.013	1507	1893	2180	3.1	3.6	4.2
median		0.048	46 887	98	1915	0.2	59 756	0.048	2237	2919	3674	4.7	6.2	7.9
U 2.5%		0.069	57 153	828	1925	1.5	66 504	0.069	3294	4522	5989	7.3	10.2	13.7

All scenarios revealed a very protracted population bottleneck period (figure 2). The median date of minimum abundance was estimated between 1915 and 1925 over all scenarios. At this point the population reached the point of maximum depletion with its minimum abundance 0.1–0.5% of pre-exploitation abundance. A second decline in abundance is also seen in the 1960s when illegal Soviet whaling was conducted (figure 2).

The models provide insight into likely values for life history parameters consistent with the historical trajectory. Examination of the model prior distributions modified by the *N*_floor_ bottleneck constraint (the ‘post-model–pre-data’ distributions, electronic supplementary material, figures S1A–P) reveals that within the uniform 0–0.12 prior range on intrinsic growth rate *R*_max_, only some *R*_max_ values were compatible with the haplotype-based constraints on minimum abundance (*N*_floor_), with *R*_max_ values over 0.09 excluded when *N*_floor_ = 36 (e.g. electronic supplementary material, figures S1D–J, S1N–P). All key prior parameters are significantly updated by the data except catch. Here, prior and posterior distributions are very similar, indicating that the measurement uncertainty incorporated into the catch scenarios is fully encompassed in the posterior estimates.

The independent check on the fit of mark recapture data to differing population abundances and trends revealed that recaptures of females supported an ROI_1995–2009_ of 0.07 (top 1% likelihood range = 0.05–0.08) and *N*_1995_ = 1104 (top 1% likelihood range = 942–1313, electronic supplementary material, figure S2A), values similar in range to estimates made by the POPAN open population model [29]. In contrast, male recaptures favoured very high ROI, with maximum likelihood values of ROI_1995–2009_ = 0.12 (range = 0.10–0.12 for the top 1% of likelihood values) and a low *N*_1995_ = 520 (*N*_1995_ top 1% likelihood range 460–660) (electronic supplementary material, figure S2B). This is likely to be an unrealistically high rate of increase for southern right whales, given their two to three-year calving cycle and long juvenile stage [20]. Maximum likelihood mark recapture abundance values for 1995 to 2009 were plotted against posterior median trajectories generated by all scenarios (electronic supplementary material, figure S3). For both datasets the recapture trend was most closely concordant with the scenario of no *N*_floor_ constraint.

A similar pattern was seen in the assessment models for the two recapture datasets; when no minimum constraint (*N*_floor_) was imposed on bottleneck size (electronic supplementary material, table S1), median ROI_1995–2009_ were 0.07 (95% probability interval PI = 0.01–0.12) and 0.10 (95% PI = 0.06–0.12) for female and male recaptures respectively, a difference of 3% annual growth per annum, although with broadly overlapping 95% PI (table 2; electronic supplementary material, table S2). Consequently pre-exploitation abundance *K* was estimated to be approximately 3000 higher with the female recaptures compared to male recaptures, and estimated *N*_min_ was also 3 times higher.

When trends were alternatively fitted using abundance values from a POPAN model, median population growth was approximately 0.05 over both catch and *N*_floor_ constraint scenarios, and estimated *K* was 28 000–29 000 for the low catch NZ and 45 500–47 000 for the high Southwest Pacific catch scenario (table 2; electronic supplementary material, table S1). These assessment scenarios were the least influenced by the bottleneck constraint, with posterior ROI_1995–2009_ estimates reduced by only 0.3% from 0.051 to 0.048 when *N*_floor_ = 36 was imposed, and median posterior *N*_min_ estimates of 75–100, regardless of bottleneck constraint.

Considering the results across all scenarios, the status of the New Zealand right whale population is currently 5–12% recovered relative to pre-exploitation abundance in 1770.

## 4. Discussion

Here we have combined contemporary genetic and demographic information with historical whaling records to reconstruct the historical demography of the southern right whale in New Zealand waters. This assessment is temporally resolved, revealing for the New Zealand region a very rapid decline in abundance over two decades, a narrow bottleneck of less than 120 whales, and a current recovery level (7–12%) that is roughly one half that estimated by the IWC's circumpolar assessment (15–20%).

The population underwent a severe decline in abundance and a very protracted exploitation-driven bottleneck in abundance under all scenarios considered. The minimum abundance of New Zealand right whales occurred in the early 20th century during a period of approximately 60 years when the population size was less than 200 (1890–1950), with minimum abundance estimated at around 110 whales in total in 1915 (figure 2). This probably represents only 25–30 mature females, consistent with the destructive nature of a hunt focusing on females with dependent calves [47]. This period coincides with continued opportunistic hunting, primarily at coastal whaling stations representing key winter calving areas, subsequent to the initial collapse of the population from the early 19th century hunts. The continual pressure at such critical habitats was a key driver of the prolonged bottleneck. From such a low level, recovery would naturally be extremely slow. Even under the most conservative catch scenario, the New Zealand population currently stands at less than 15% of pre-whaling abundance. Southern right whales have been protected from commercial whaling for more than 80 years, but illegal Soviet whaling of 372 whales from the southwest Pacific in the 1960s [30] has inevitably also delayed recovery. When catches off southeast Australia are allocated to the New Zealand population (the ‘Southwest Pacific’ catch series), median recovery levels are less than 9%. Based on the *N*_floor_ = 36 assumption in the population model we developed, the population will not recover to 95% of its carrying capacity for at least 50 years. However, it is possible that while the population has been at low abundance the ecosystem has changed in unknown ways, making such projections uncertain.

Despite the low level of recovery, reasonable rates of increase have been reported from the sub-Antarctic Auckland Islands, around 7% [29]. Multiple lines of evidence connect the recovering Auckland Islands southern right whale population with those sighted off mainland New Zealand [28], suggesting that a component of the sub-Antarctic population is re-colonizing the New Zealand bays where these whales were formerly very abundant. Whether the increasing trend seen in the sub-Antarctic islands is mirrored in New Zealand waters is not yet known.

Offshore and coastal whaling for the New Zealand right whale in the mid-19th century was ‘one of the most extensive, prolonged, and thorough campaigns of wildlife exploitation in all of human history’ ([11, p. 41]). While the depletion of the North Atlantic right whale occurred over many centuries, in New Zealand the most intense period of the fishery spanned 50 years (figure 2). The mobility of offshore whalers also meant that the rate of decline in abundance was probably much greater, and the period of most intense whaling much briefer, than it subsequently was in the North Pacific [48,49]. Over the 1830s and 1840s, for example, the population of New Zealand right whales was reduced by roughly 90% of its pre-whaling abundance of approximately 29 000 whales.

### 4.1. How many whales before whaling?

Prior to this study, the recovery levels of southern right whales have only been assessed at the species level [14,50]. Although understandable, such simplifications limit our understanding of population response to different histories of regional exploitation and recovery. A previous species-level assessment of southern right whales concluded that circumpolar abundance prior to whaling numbered around 60 000 [14], although with adjustments for struck and lost rates and a *N*_floor_ constraint the model estimate increased to 100 000 [38]. The genetic-based estimate of long-term global abundance for southern right whales (converting a measurement of species diversity, *θ*) is however more than double this number, at 202 000–370 500 whales [38]. This disparity may be caused by population structuring within the species, which inflates measured diversity. However, disparities in genetic and demographic based abundance estimates also exist for other species, including humpback whales [51] and gray whales [52]; in both cases genetic estimates of long-term abundance are much larger than estimates based on population dynamics and catch histories, and suggest that either the methods of measuring abundance are incorrect, or that these Northern Hemisphere whale populations were much larger in the Quaternary (the time-frame of genetic estimates) than they were in the century preceding exploitation (the time frame of the population models).

Interestingly, we find good concordance between genetic and demographic based estimates of pre-exploitation abundance for the New Zealand southern right whale population. Genetic-based estimates provide a long-term abundance estimate of approximately 35 000 whales [17], which falls between the pre-exploitation numbers yielded by the ‘NZ-only’ (28 000–33 000) and ‘Southwest Pacific’ (43 000–47 000) catch scenarios explored in this study. The genetic estimate reflects long-term population abundance over a Quaternary timescale (approx. 5000–1 million years) suggesting that average abundance of southern right whales in the southwest Pacific marine ecosystem through the late Quaternary was of similar magnitude to their abundance prior to 19th century whaling.

These data suggest right whales have been abundant in New Zealand near-shore waters for millennia, and are therefore likely to have played a significant role in the marine food web, both in terms of offshore consumption of zooplankton (e.g. *Neocalanus tonsus* [53]) and near-shore nutrient enrichment of coastal embayments through defecation [54].

### 4.2. Population structure uncertainty

In this study the problem of past catch allocation has been addressed by the development of a ‘high catch’ case for the population, which assumes that whales caught off southeastern Australia originated from the New Zealand right whale population. Where population limits are not well known it is also possible to model multiple populations simultaneously, so that catches from localities where the populations mix can be allocated back to source populations in a density-dependent fashion. This has been implemented for many Southern Hemisphere humpback breeding populations, which seasonally mix on Southern Ocean feeding grounds and were extensively hunted there [55]. With in-depth reconstruction of other regional catch histories for southern right whale populations, it will be possible to model multiple populations simultaneously, providing an additional perspective on past numbers and regional catch impacts. Further information gathering on early catches and the extent of bay whaling around Australia would enable multi-stock modelling of population trajectories across New Zealand and Australia. Other biological data besides genetics can also be applied to delimit populations; for example species such as blue and fin whales show acoustic variation in their vocalizations between oceans, and these can be used to infer seasonal movements and structuring of populations [56,57]. In an assessment context, passive acoustic data on the seasonal distribution of blue whale call types in the North Pacific were recently used to assign past catches to North Pacific blue whale populations, by season and geographic area [58]. Balaenids (right and bowhead whales) are not known to show population-level variation in their calls [59] but passive acoustic detection of seasonal presence (e.g. on former whaling grounds such as the Kermadec Islands, figure 1) would be very useful for understanding the offshore distribution of these recovering populations and also validating optimum habitat models constructed from whaling data [53].

### 4.3. Integrating mark-resighting data

A sample of individual capture histories from the New Zealand population, informative about recent abundance and trend, was fitted directly into the model likelihood, applying a technique recently developed to measure the modern whaling impact on humpback whales [10]. This provides a single statistical treatment for the capture histories within the assessment model. Without this integration, mark recapture data are used to generate measurements of abundance and trend independently, and those measurements are then fed into the population assessment model, with generalized variance measures that do not necessarily reflect true uncertainty. In this study, both approaches to measuring abundance and trend were explored.

The ‘super-population’ abundance of the New Zealand southern right whales was estimated in a 2009 genotype capture–recapture survey to be 2200 whales with population growth estimated at 7% per annum (95% CI 5%–9%) [29]. The ‘super-population’ is the total number of individuals that enter the focal population between the first and last survey occasions. This estimate was made using sex-specific POPAN models for males and females using recapture data from eight years of field surveys from 1995–2009. The female POPAN model was modified to account for capture heterogeneity between years in reproductive females and total abundance was estimated by combining data from the sex-specific models.

In our study, male and female recaptures were used to fit population abundance and trend separately, with abundance doubled to account for both sexes in both cases. Our results showed a substantial impact of different mark recapture datasets on estimated trend, highlighting the importance of checking fixed assumptions within the mark recapture model. Apparent annual survival of males (a combination of survival and fidelity) was estimated at approximately 0.84 using the POPAN open population model compared to approximately 1.00 for females, suggesting male southern right whales show lower site fidelity to the Auckland Islands calving ground [29]. When male resightings were combined with a fixed annual survival rate of 0.97 in the ‘closed’ population framework of our assessment model, estimated population growth became very high to fit the low number of resights in the data (a simple forward simulation with annual survival fixed to 0.84 gave ROI values closer to 7%; data not shown). Population closure assumptions are not violated for females over the 15 year study period, so these data are considered more appropriate for the assessment model. However female recaptures within the assessment model yielded higher 2009 abundance estimates (approx. 2750 whales) than those measured in the open population framework (approx. 2200 whales), which accommodates capture heterogeneity due to calving. The open population analysis showed that inter-annual capture heterogeneity within the female dataset could positively bias abundance estimates if not explicitly accommodated in the mark recapture model, which may be a factor influencing these high estimates [29]. Further development of the integrated capture–recapture model to accommodate stage-specific (in this case calving-related) capture heterogeneity and relax some assumptions of population closure could improve the flexibility of this assessment structure in the future.

### 4.4. Population modelling and density dependence

This population reconstruction of the New Zealand southern right whales represents the first regional assessment of a southern right whale population, directly integrating estimates of past catches and their associated variance over a 200-year time frame, and also incorporating genetically derived information on minimum abundance.

Our modelling was based on assumptions of a density-dependent decline in population growth *r* on approach to carrying capacity *K*, with *R*_max_ and *K* kept constant over the population history. These are standard procedure for population assessments conducted by the International Whaling Commission. The density-dependent model used in this study favours population growth rates below 8% since higher rates are associated with low bottleneck abundance. A recent assessment of alternative population growth models has suggested that baleen whale population growth may be better explained by a selection-delayed model of recovery [4], whereby population growth is not fixed and is mediated by density-dependent competitive interactions among individuals, i.e. intra-specific natural selection. Applying this model to the southern right whales at the circumpolar level has yielded much higher estimates of pre-exploitation abundance than under the density-dependent models (approx. 145 000 whales compared to approx. 85 000 whales) [4]. Application of this alternative model structure might similarly increase levels of pre-exploitation abundance for New Zealand whales, since this population is contained within the circumpolar species trajectory, and mirrors the broader pattern, in that the New Zealand population is currently at low levels relative to pre-exploitation numbers, and was subject to a prolonged population bottleneck.

Assumptions of constant *K* through time may also be violated if the extent of available calving and foraging habitats have changed significantly over the past 200 years, due to either environmental shifts or shifts in available niche space due to intra- or inter-species competition. For example, genetic diversity based estimates of long-term abundance suggest more than 20 000 southern right whales were using the Victoria calving grounds (SE Australia) in the past, compared to 35 000 off New Zealand [17]. Since there are less than 400 right whales sighted in southeast Australia today, the loss of a large local calving ground could potentially have increased available foraging habitat for New Zealand right whales. Population assessment of the catch histories, pre-exploitation abundance and recovery of calving grounds around Australia would be required to evaluate these shifts with accuracy.

### 4.5. Estimating bottleneck abundance *N*_min_

Over 80% of all right whale catches were made between 1830 and 1850, driving the population to extremely low abundance [9]. Our assessment indicates that the New Zealand population has since been recovering slowly from a minimum abundance (or bottleneck) point about a century ago. In order to add some biological realism to the bottleneck point, we constructed our primary assessment scenarios with a minimum constraint on bottleneck abundance (*N*_floor_) of 36. This was derived by multiplying current haplotype richness (*h* = 12) in the New Zealand population by three, assuming each haplotype represents a single adult female that survived the bottleneck and scaling to account for males and sub-adults. However it is important to note that imposing an *N*_floor_ constraint on the population model, as applied here and in recent population assessments of humpback whales [55], updates the model priors independently of the assessment data, because high *R*_max_ values are associated with very small bottleneck abundance values, *N*_min_. Since *N*_floor_ has no likelihood component, formally this model structure is statistically invalid.

Developing soft priors on bottleneck size *N*_min_ rather than a lower bound constraint in future models will be a useful improvement to the model structure. Such a prior will be non-trivial to develop; previous investigations have made upward corrections from current haplotype frequencies to provide a minimum bound for *N*_min_ [38] where a single population trajectory can be assumed (i.e. with no immigration/emigration components), and these could be further developed to form a soft prior. Since most whale populations exhibit a degree of inter-population connectivity (exchanging migrants with neighbouring breeding grounds), detailed consideration of inter-population connectivity (i.e. genetic contribution of immigrants to current haplotype frequencies) subsequent to the bottleneck period will be required to develop a bottleneck prior that is broadly useful, supported with individual based population modelling to evaluate the performance of this prior in a variety of simulated conditions.

In this study, the *N*_floor_ constraint increased *N*_min_ and reduced the rate of population increase to 0.04–0.05, producing values much lower than that estimated independently with POPAN open models, close to the lower 95% boundary of the POPAN growth estimate of 0.05 [29]. With no bottleneck constraint, 35–90 individuals were estimated (roughly 10–30 adult females). With the *N*_floor_ = 36 constraint, estimated *N*_min_ spans 90–120, implying 30–40 adult females in the bottleneck population. This latter is low relative to current observed haplotype richness (*h* = 12), but not inconceivable. Given that approximately 100 years has elapsed since the bottleneck, and low inter-annual site fidelity is measured for males [29], it is possible that two to three of the current low-frequency haplotypes have been introduced to the population through immigration since the bottleneck point; for example two of the 12 observed haplotypes have only been identified in males. However even with a slight reduction in haplotype numbers crossing the bottleneck, *N*_floor_ = 36 remains a reasonable conservative constraint on minimum bottleneck abundance; even if less than 12 haplotypes survived the population bottleneck, the current constraint assumes that only one adult female with each haplotype was present at the bottleneck, which is unrealistic and therefore conservatively low. Our findings reveal that the current density-dependent model structure does not easily accommodate both a 7% ROI on the calving ground, and a bottleneck abundance of more than 50 individuals. Methodological developments to include a prior on *N*_min_ and to relax the standard assumptions of density dependence (e.g. [4]) will be useful for constructing future assessments of this population.

### 4.6. ‘A most destructive fishery’ ^1^

The population dynamics model used in this study makes no assumption about the impact of sex bias in catches, though, in reality, practices such as bay whaling specifically targeted mothers and calves [47], since these frequented protected coastal waters during winter. For example, this was so much the focus of the South African bay whaling campaign that over 75% of the whales taken were estimated to have been female [60]. If catches were female-biased, population declines could become more pronounced because reproductive rates are more dependent on the numbers of females than males (e.g. [61]). For the New Zealand southern right whales, the majority of bay and shore based whaling took place in the 1840s, driving the 95% decline in right whale abundance. If the remaining population was skewed towards males, population growth rates were likely to have been low as a consequence of the lack of available females. It is difficult to quantify the impact, however, as information on the age and sex structure of historical catches is unknown; most catch reconstruction has been done by converting barrels of landed and exported oil into whales [9]. Whaling logbooks may be somewhat useful, as in a few cases sightings of adult and dependent whales can be identified as mothers and calves. However the sex of other landed whales went unrecorded. With further analyses of the exploitation history and available life history data, development of age and sex-structured models for the population can allow such harvesting scenarios to be explored in more detail.

## 5. Conclusion

Pre-modern whaling had a devastating impact on southern right whale populations, and here we have quantified for the first time the historical impact at a population level, reconstructing the population trajectory for the New Zealand right whale. We find that the population declined rapidly following early 19th century whaling, and came close to extinction early in the 20th century, with less than 40 mature females estimated as surviving the bottleneck. The assessment outcomes of this study in terms of population status and pre-exploitation abundance are consistent over multiple catch history, mark recapture data and bottleneck constraint scenarios. These catch history scenarios show that the current population is still at less than 12% of its pre-exploitation abundance, which is estimated at over 28 000 whales.

## Supplementary Material

Text S1 describes the population model construction in detail. Text S2 describes how the prior distribution for each catch history was constructed.

## Supplementary Material

Text Supplement S2

## Supplementary Material

Table S1 Key biological parameters estimated for the New Zealand southern right whale over ‘catch maximum’ and ‘catch minimum’ population modelling scenarios, with no Nfloor constraint imposed (male and female recaptures and population model fitted to relative abundance). Table S2 shows posterior medians and 95% probability intervals for key biological parameters estimated for the NZ southern right whale over six population modelling scenarios (male recaptures) Figs. S1A-P show the posterior and modified prior (post-model, pre-data) distributions on key biological parameters for all scenarios Fig. S2 shows the N1995 abundance and population growth (R) values corresponding to the top 1% of likelihood scores for (A) female and (B) male recaptures. Fig. S3 plots the posterior median abundance values between 1995-2009 for (A) female and (B) male recaptures of southern right whales for all scenarios explored in this study.

## Footnotes

1 p. 100, from ‘Minutes of evidence taken before the Select Committee on State of Colony of New Zealand’, British Parliamentary Paper 1844 (556).

## Data accessibility

The R code used in all population modelling can be found on DataDryad: http://dx.doi.org/10.5061/dryad.c3s62.

Electronic supplementary material details are as follows:

Text S1 describes the population model construction in detail.
Text S2 describes how the prior distribution for each catch history was constructed.
Table S1 shows key biological parameters estimated for the New Zealand southern right whale over ‘catch maximum’ and ‘catch minimum’ population modelling scenarios, with no *N*_floor_ constraint imposed (male and female recaptures and population model fitted to relative abundance).
Table S2 shows posterior medians and 95% probability intervals for key biological parameters estimated for the NZ southern right whale over six population modelling scenarios (male recaptures).
Figure S1A–P shows the posterior and modified prior (post-model, pre-data) distributions on key biological parameters for all scenarios.
Figure S2 shows the *N*_1995_ abundance and population growth (*R*) values corresponding to the top 1% of likelihood scores for (A) female and (B) male recaptures.
Figure S3 plots the posterior median abundance values between 1995 and 2009 for (A) female and (B) male recaptures of southern right whales for all scenarios explored in this study.
