# Supplementary material for: An integrated approach to historical population assessment of the great whales: case of the New Zealand southern right whale
Source: R Soc Open Sci. 2016 Mar 16;3(3):150669. doi: 10.1098/rsos.150669 (PMC4821268; doi:10.1098/rsos.150669)
Supplement: Text S1 describes the population model construction in detail. Text S2 describes how the prior distribution for each catch history was constructed. [file rsos150669supp1.docx]

**Text Supplement S1**

*Population model construction*

Mark recapture data form a matrix comprising counts of animals seen in a specific year and re-seen in a subsequent year. If a whale is re-seen a second time, the first re-sighting is treated as a new sighting that is first re-seen at the second re-sighting.

This information is incorporated into the model likelihood as follows:

Two parameters are obtained from the mark recapture data:

*n_y_* , the number of animals captured in year *y,* and

*m_y’,y_* , the number of animals captured in year *y* that were recaptured in year *y’.*

If *p_y_* is the probability that an animal is seen in a region in year *y,* then the number of animals captured in year *y* is given by:

*n_y_* = *p_y_ N_y_*  (1)

where *N_y_* is the total (1+) population. The model predicted number of animals captured in year *y* that were recaptured in year *y’* is given by:

$\hat{m}_{y',y}=p_{y}p_{y'}N_{y}e^{-M(y^{'}-y)}$ (2)

*M* is the natural mortality rate (set here to equal 0.03 yr^-1^). This is similar to survival rates measured for the southern right whale population wintering off South Africa ([Best et al. 2001](#_ENREF_1)).

The probability of model-predicted $\hat{m}_{y',y}$*,* given the observed $m_{y',y}$ is determined assuming a Poisson distribution. This is an approximation to the multinomial distribution used in standard mark recapture analyses, which is applicable here because annual capture probabilities are small.

The contribution of this prediction to the likelihood is as follows:

$\frac{{(\hat{m}}_{y,y^{'}})^{m_{y-y'}}}{\hat{m}_{y^{'},y}!}e^{{-\hat{m}}_{y',y}}$ (3)

The negative of the log likelihood component for the capture recapture data is then:

$-\ln L= \sum_{y=y_{0}}^{y_{f}-1} \sum_{y^{'}=y+1}^{y_{f}} [-m_{y',y}\ln\hat{m}_{y',y}+ \hat{m}_{y',y}$] (4)

where *y_0_* is the first year of captures and *y_f_* is the last year of recaptures. Minimisation occurs over the estimable parameters *r* and *N* in year *0.*

Results are generated for three priors: (i) an *r* prior of the form *r* ~ U[0,0.12] in order to bound biologically realistic increase rates for southern right whales ([Best et al. 2001](#_ENREF_1)), (ii) an uninformative prior on recent abundance (*N_2009_*) spanning 500-20,000, of the form *N* ~ U[500,20,000], (iii) a prior on catch history, accommodating uncertainty in annual catches (see Text S2).

All prior sets for which *N_min_* was below the *N_floor_* constraint were given zero likelihood.

*Sample importance resampling to form the posterior distribution*

The likelihood of each prior set *l* was summed to a cumulative likelihood value *L^c^* as prior sets accrued:

$L^{c}=l^{1}+l^{2}+ l^{3}+\ldots+l^{n}$ (5)

until *L^c^* > *t*, where *t* = a fixed threshold value. That prior set was then drawn for the posterior distribution. Draws *d* of that prior set corresponded to:

$d=floor \left( \frac{L^{c}-t}{t}+1 \right)$ (6)

at which point the new cumulative likelihood *L^c2^* becomes:

$L^{c2}= L^{c}-(d*t)$ (7)

Samples were accrued until a given number of posterior resamples were reached. In this study, the threshold *t* was set to provide roughly one resample for every 250 prior sets sampled. Thresholds were set to 3 x 10^-4^, 3 x 10^-3^ and 3 x 10^-12^ for female, male and relative/absolute abundance data fitting respectively. Two thousand posterior resamples were collected for each scenario.

Best P, Brandao A, Butterworth D (2001) Demographic parameters of southern right whales off South Africa. J Cetacean Res Manage (Special Issue) 2:161-169
