# Supplementary material for: An integrated approach to historical population assessment of the great whales: case of the New Zealand southern right whale
Source: R Soc Open Sci. 2016 Mar 16;3(3):150669. doi: 10.1098/rsos.150669 (PMC4821268; doi:10.1098/rsos.150669)
Supplement: Text Supplement S2 [file rsos150669supp2.docx]

**Text Supplement S2**

*Constructing a prior distribution for the catch history*

For every *N_current_* and *R_max_* prior selection, a unique right whale catch series was constructed following these steps:

1. American whaleboat catches off New Zealand and East Australia. Annual catch uncertainty was assumed normally distributed around mean estimates ([standard errors calculated for each series are given in Table 2, Carroll et al. 2014](#_ENREF_1)). Random sampling from each annual catch estimate was used to generate a unique American catch series for each prior sample for each locality. Annual catches were then ‘smeared’ over 6 subsequent years, according to proportions of American ships calculated to be whaling in southwest Pacific waters in those years ([Table 3, Carroll et al. 2014](#_ENREF_1)). Bay whaling catches estimated from logbooks were subtracted from this series to obtain a bay whaling and offshore series. Given uncertainty in the numbers of American ships bay whaling (see ‘*Catch History*’), two alternate bay whaling series were compiled to provide alternate scenarios of high versus low bay whaling in New Zealand waters. Concomitantly, different levels of offshore catch were also associated with each of these scenarios.
2. Right whales caught by French offshore whaling voyages ([Du Pasquier 1986](#_ENREF_2)) were similarly smeared, with right whale catches equally allocated 2, 3 and 4 years after departure from French ports ([Table 3, Carroll et al. 2014](#_ENREF_1)). During the American catch series compilation, New Zealand offshore and bay whaling operations were distinguished using ships logbooks. However catch locations and operation types (offshore, bay whaling) were not distinguished for French pelagic whaling catches ([Du Pasquier 1986](#_ENREF_2)). The French catches were therefore assumed to mirror those of the American whaling catches in terms of their relative frequencies of whaling in; (i) New Zealand offshore, (ii) East Australian offshore, and (iii) New Zealand coastal (bay whaling) operations. The smeared French series was thus divided up to generate three sub-series using the relative catch series’ generated in (1).
3. The American and French offshore catch series’ for New Zealand and East Australia were each summed to provide total offshore catch series’ for each region.
4. American and French bay whaling catch series for New Zealand (see 2) were summed to provide total bay whaling catches.
5. Two coastal catch series were generated for New Zealand; one which included the two ‘low case’ series’ for coastal and bay whaling, and the other which included the two ‘high case’ series. Coastal catches from Tasmania and New South Wales were used for the coastal southeast Australia catch series.
6. The total coastal catch series for New Zealand was made by summing (4) and (5) above, while the southeast Australia coastal catch series was represented by (5).
7. Following Carroll et al. (2014), upward corrections were added for whales struck but lost during coastal and offshore whaling. For the coastal series (6), each annual catch was corrected upward by a factor randomly picked from a normal prior distribution with mean=1.26 and standard deviation (SD) = 0.05. For the offshore series (3), catches were corrected upward by a factor randomly picked from a normal prior distribution with mean=1.45, SD=0.054, reflecting the struck and lost rates estimated from these types of whaling.
8. The offshore and coastal series were combined. Additional catches from Soviet modern whaling were added (no correction for whales struck but lost is required as modern whaling was efficient). The majority of Soviet catches were taken close to the Auckland islands and assumed to belong to the New Zealand stock. A further 78 were killed south of Tasmania and therefore associated with southeast Australia ([Tormosov et al. 1998](#_ENREF_3)).

Carroll EL, Jackson JA, Paton D, Smith TD (2014) Two Intense Decades of 19th Century Whaling Precipitated Rapid Decline of Right Whales around New Zealand and East Australia. PLoS One 9:e93789

Du Pasquier T (1986) Catch history of French right whaling mainly in the South Atlantic. Report of the International Whaling Commission (Special Issue) 10:269-274

Tormosov DD, Mikhaliev YA, Best PB, Zemsky VA, Sekiguchi K, Brownell Jr RL (1998) Soviet catches of Southern Right Whales *Eubalaena australis,* 1951-1971. Biological data and conservation implications. Biol Conserv 86:185-197
