# Supplementary material for: An integrated approach to historical population assessment of the great whales: case of the New Zealand southern right whale
Source: R Soc Open Sci. 2016 Mar 16;3(3):150669. doi: 10.1098/rsos.150669 (PMC4821268; doi:10.1098/rsos.150669)
Supplement: Table S1 Key biological parameters estimated for the New Zealand southern right whale over ‘catch maximum’ and ‘catch minimum’ population modelling scenarios, with no Nfloor constraint imposed (male and female recaptures and population model fitted to relative abundance). Table S2 shows posterior me [file rsos150669supp3.docx]

**Electronic Supplement Figs., S1-3B**

Figure S1A-P show the (i) post-model pre-data (blue) and (ii) posterior (pink) distributions of key biological parameters for each population assessment scenario, as numbered in Table 1. N2009, N2015 and N2020 refer to abundance in 2009, 2015 and 2020 respectively. *R_max_* refers to the maximum intrinsic growth rate of the population, *N_min_* refers to bottleneck abundance, ‘Status 2015’ and ‘Status 2020’ refer to population recovery levels as a proportion of pre-exploitation abundance (carrying capacity, also known as *K*) in 2015 and 2020 respectively.

Fig. S1A. Scenario 1: Low case NZ catches, female recaptures, no *N_floor_* constraint

Fig. S1B. Scenario 2: Low case NZ catches, male recaptures, no *N_floor_* constraint

Fig. S1C. Scenario 3: Low case NZ catches, fitted to POPAN relative abundance and 2009 absolute abundance, no *N_floor_* constraint

Fig. S1D. Scenario 4: Low case NZ catches, female recaptures, *N_floor_* constraint = 36

Fig. S1E. Scenario 5: Low case NZ catches, male recaptures, *N_floor_* constraint = 36

Fig. S1F. Scenario 6: Low case NZ catches, fitted to POPAN relative abundance and 2009 absolute abundance, *N_floor_* constraint = 36

Fig. S1G. Scenario 7: High case NZ catches, female recaptures, *N_floor_* constraint = 36

Fig. S1H. Scenario 8: High case NZ catches, male recaptures, *N_floor_* constraint = 36

Fig. S1I. Scenario 9: Low case Southwest Pacific catches, female recaptures, *N_floor_* constraint = 36

Fig. S1J. Scenario 10: Low case Southwest Pacific catches, male recaptures, *N_floor_* constraint = 36

 Fig. S1K. Scenario 11: High case Southwest Pacific catches, female recaptures, no *N_floor_* constraint

Fig. S1L. Scenario 12: High case Southwest Pacific catches, male recaptures, no *N_floor_* constraint

Fig. S1M. Scenario 13: High case Southwest Pacific catches, fitted to POPAN relative abundance and 2009 absolute abundance, no *N_floor_* constraint

Fig. S1N. Scenario 14: High case Southwest Pacific catches, female recaptures, *N_floor_* constraint = 36

Fig. S1O. Scenario 15: High case Southwest Pacific catches, male recaptures, *N_floor_* constraint = 36

 Fig. S1P. Scenario 16: High case Southwest Pacific catches, fitted to POPAN relative abundance and 2009 absolute abundance, *N_floor_* constraint=36

Fig. S2 plots the *N_1995_* abundance and population growth (*R*) values corresponding to the top 1% of likelihood scores for (A) female and (B) male recaptures.

Fig. S3. Plot of (A) female and (B) male recaptures of southern right whales from 1995-2009 alongside the posterior median abundance values for all scenarios explored in this study. Two scenarios where the assessment has been directly fitted to POPAN-based relative and absolute abundance estimates are also indicated as ‘POPAN’ trends (Carroll et al. 2013).

Table S1. Key biological parameters estimated for the New Zealand southern right whale over ‘catch maximum’ and ‘catch minimum’ population modelling scenarios, with no *N_floor_* constraint imposed (1000 resamples). *K* refers to pre-exploitation abundance in 1829, *N_min_* the estimated minimum bottleneck abundance, *ROI* the rate of annual population increase, *R_max_* the intrinsic rate of population growth. ‘% Recovery status’ and ‘Max % depletion’ show abundance relative to *K,* in a given year and *N_min_* year, respectively.

| Scenario | Case | *R_max_* | *K* | *N_min_* | *N_min_* year | Max % depletion | Total Catch | *ROI*_1995-2009_ | Abundance | | | % Recovery status | | |
| --- | --- | --- | --- | --- | --- | --- | --- | --- | --- | --- | --- | --- | --- | --- |
|  |  |  |  |  |  |  |  |  | N_2009_ | N_2015_ | N_2020_ | 2009 | 2015 | 2020 |
| Females | | |  |  |  |  |  |  |  |  |  |  |  |  |
| NZ only | Low |  |  |  |  |  |  |  |  |  |  |  |  |  |
| L 2.5% |  | 0.009 | 20522 | 7 | 1897 | 0.0 | 29344 | 0.009 | 2155 | 2494 | 2697 | 6.7 | 7.4 | 7.8 |
| Median |  | 0.068 | 27125 | 38 | 1925 | 0.1 | 35619 | 0.068 | 2866 | 4250 | 5885 | 10.6 | 15.8 | 21.9 |
| U 2.5% |  | 0.115 | 36329 | 1307 | 1930 | 3.7 | 42154 | 0.114 | 3833 | 6693 | 10784 | 16.4 | 29.2 | 47.2 |
| NZEA | High |  |  |  |  |  |  |  |  |  |  |  |  |  |
| L 2.5% |  | 0.007 | 34702 | 8 | 1901 | 0.0 | 53551 | 0.007 | 2176 | 2527 | 2661 | 4.1 | 4.4 | 4.6 |
| Median |  | 0.066 | 43262 | 46 | 1925 | 0.1 | 59580 | 0.066 | 2864 | 4173 | 5755 | 6.6 | 9.7 | 13.5 |
| U 2.5% |  | 0.114 | 58353 | 1640 | 1930 | 2.9 | 66549 | 0.114 | 3919 | 6805 | 10995 | 10.0 | 18.3 | 29.7 |
| Males | | |  |  |  |  |  |  |  |  |  |  |  |  |
| NZ only | Low |  |  |  |  |  |  |  |  |  |  |  |  |  |
| L 2.5% |  | 0.064 | 19161 | 6 | 1925 | 0.0 | 29488 | 0.064 | 2033 | 3148 | 4352 | 0.07 | 0.11 | 0.16 |
| Median |  | 0.103 | 24282 | 10 | 1930 | 0.0 | 35367 | 0.103 | 2623 | 4674 | 7524 | 0.11 | 0.20 | 0.31 |
| U 2.5% |  | 0.119 | 30539 | 44 | 1930 | 0.2 | 42003 | 0.119 | 3580 | 6604 | 10801 | 0.16 | 0.29 | 0.47 |
| NZEA | High | |  |  |  |  |  |  |  |  |  |  |  |  |
| L 2.5% |  | 0.062 | 33281 | 7 | 1925 | 0.0 | 53862 | 0.062 | 1972 | 3028 | 4153 | 0.05 | 0.07 | 0.10 |
| Median |  | 0.103 | 38632 | 12 | 1929 | 0.0 | 59762 | 0.103 | 2634 | 4671 | 7555 | 0.07 | 0.12 | 0.20 |
| U 2.5% |  | 0.119 | 45841 | 51 | 1930 | 0.1 | 66435 | 0.119 | 3529 | 6612 | 11167 | 0.10 | 0.18 | 0.31 |
| Fitted POPAN trend | | |  |  |  |  |  |  |  |  |  |  |  |  |
| NZ only | Low |  |  |  |  |  |  |  |  |  |  |  |  |  |
| L 2.5% |  | 0.018 | 22153 | 18 | 1897 | 0.1 | 29434 | 0.018 | 1530 | 1966 | 2259 | 5.1 | 6.2 | 7.0 |
| Median |  | 0.051 | 28191 | 78 | 1921 | 0.3 | 35439 | 0.051 | 2232 | 3011 | 3865 | 7.9 | 10.7 | 13.7 |
| U 2.5% |  | 0.087 | 35404 | 559 | 1926 | 1.8 | 41899 | 0.087 | 3212 | 4582 | 6444 | 12.4 | 18.4 | 26.0 |
| NZEA | High |  |  |  |  |  |  |  |  |  |  |  |  |  |
| L 2.5% |  | 0.017 | 37868 | 20 | 1901 | 0.0 | 53321 | 0.017 | 1526 | 1959 | 2276 | 3.1 | 3.9 | 4.4 |
| Median |  | 0.051 | 45793 | 85 | 1915 | 0.2 | 59548 | 0.051 | 2233 | 3006 | 3840 | 4.8 | 6.5 | 8.4 |
| U 2.5% |  | 0.086 | 55871 | 632 | 1926 | 1.1 | 66322 | 0.086 | 3272 | 4565 | 6557 | 7.4 | 11.1 | 15.9 |

Table S2. Male recaptures for *N_floor_* = 36

| Scenario | Case | *R_max_* | *K* | *N_min_* | *N_min_* year | Max % depletion | Total Catch | *ROI*_1995-2009_ | Abundance | | | % Recovery status | | |
| --- | --- | --- | --- | --- | --- | --- | --- | --- | --- | --- | --- | --- | --- | --- |
|  |  |  |  |  |  |  |  |  | N_2009_ | N_2015_ | N_2020_ | 2009 | 2015 | 2020 |
| N_floor_=36 |  |  |  |  |  |  |  |  |  |  |  |  |  |  |
| NZ only | Low |  |  |  |  |  |  |  |  |  |  |  |  |  |
| L 2.5% |  | 0.031 | 21979 | 36 | 1914 | 0.1 | 29457 | 0.031 | 1773 | 2338 | 2824 | 5.9 | 7.6 | 9.1 |
| Median |  | 0.060 | 27697 | 52 | 1925 | 0.2 | 35603 | 0.060 | 2333 | 3265 | 4343 | 8.4 | 11.9 | 15.8 |
| U 2.5% |  | 0.069 | 34369 | 224 | 1926 | 0.7 | 42256 | 0.069 | 3154 | 4558 | 6202 | 12.5 | 18.0 | 24.5 |
| NZ only | High |  |  |  |  |  |  |  |  |  |  |  |  |  |
| L 2.5% |  | 0.030 | 25312 | 37 | 1914 | 0.1 | 34119 | 0.030 | 1787 | 2297 | 2857 | 5.2 | 6.6 | 7.9 |
| Median |  | 0.060 | 30942 | 53 | 1925 | 0.2 | 40225 | 0.060 | 2301 | 3147 | 4087 | 7.4 | 10.4 | 13.9 |
| U 2.5% |  | 0.069 | 37753 | 251 | 1926 | 0.7 | 47161 | 0.068 | 3088 | 4266 | 5680 | 10.7 | 15.5 | 21.2 |
| NZEA | Low |  |  |  |  |  |  |  |  |  |  |  |  |  |
| L 2.5% |  | 0.032 | 34922 | 37 | 1914 | 0.0 | 48546 | 0.032 | 1790 | 2357 | 2854 | 1.9 | 2.4 | 2.7 |
| Median |  | 0.062 | 40859 | 53 | 1925 | 0.1 | 54825 | 0.063 | 2347 | 3333 | 4473 | 3.2 | 5.1 | 7.7 |
| U 2.5% |  | 0.071 | 48563 | 234 | 1926 | 0.4 | 61632 | 0.071 | 3117 | 4591 | 6350 | 4.6 | 8.6 | 14.6 |
| NZEA | High |  |  |  |  |  |  |  |  |  |  |  |  |  |
| L 2.5% |  | 0.032 | 38460 | 36 | 1914 | 0.1 | 53513 | 0.032 | 1803 | 2369 | 2846 | 3.8 | 4.7 | 5.7 |
| Median |  | 0.063 | 44228 | 50 | 1925 | 0.1 | 59599 | 0.063 | 2351 | 3350 | 4539 | 5.3 | 7.7 | 10.3 |
| U 2.5% |  | 0.071 | 51763 | 231 | 1926 | 0.4 | 66480 | 0.071 | 3191 | 4690 | 6515 | 7.6 | 11.2 | 15.8 |

*K* refers to pre-exploitation abundance in 1829, *N_min_* the estimated minimum bottleneck abundance, *ROI* the rate of annual population increase, and *R_max_* the intrinsic rate of population growth. ‘Max % Depletion’ refers to the minimum abundance of this population during it’s exploitation history, relative to pre-exploitation abundance. ‘% Recovery status’ and ‘Max % depletion’ show abundance as a proportion of pre-exploitation abundance *K,* in a given year and *N_min_* year, respectively.
